# Development of a Multiplex Loop-Mediated Isothermal Amplification (LAMP) Method for Simultaneous Detection of Spotted Fever Group Rickettsiae and Malaria Parasites by Dipstick DNA Chromatography

**DOI:** 10.3390/diagnostics10110897

**Published:** 2020-11-02

**Authors:** Lavel Chinyama Moonga, Kyoko Hayashida, Naoko Kawai, Ryo Nakao, Chihiro Sugimoto, Boniface Namangala, Junya Yamagishi

**Affiliations:** 1 Division of collaboration and education, Research Center for Zoonosis Control, Hokkaido University, Sapporo 001-0020, Hokkaido, Japan; lavelmwanga@gmail.com (L.C.M.); birdman0424@gmail.com (N.K.); sugimoto@czc.hokudai.ac.jp (C.S.); junya@czc.hokudai.ac.jp (J.Y.); 2 Laboratory of Parasitology, Graduate School of Infectious Diseases, Faculty of Veterinary Medicine, Hokkaido University, Kita-18, Nishi-9, Sapporo 060-0818, Hokkaido, Japan; ryo.nakao@vetmed.hokudai.ac.jp; 3 Department of Paraclinical Studies, School of Veterinary Medicine, University of Zambia, P.O. Box 32379, Lusaka 10101, Zambia; b.namangala@unza.zm

**Keywords:** spotted fever group rickettsia, SFG rickettsiosis, malaria, multiplex detection, loop-mediated isothermal amplification (LAMP), dipstick DNA chromatography

## Abstract

Spotted fever group (SFG) rickettsiae causes febrile illness in humans worldwide. Since SFG rickettsiosis’s clinical presentation is nonspecific, it is frequently misdiagnosed as other febrile diseases, especially malaria, and complicates proper treatment. Aiming at rapid, simple, and simultaneous detection of SFG *Rickettsia* spp. and *Plasmodium* spp., we developed a novel multiple pathogen detection system by combining a loop-mediated isothermal amplification (LAMP) method and dipstick DNA chromatography technology. Two primer sets detecting SFG *Rickettsia* spp. and *Plasmodium* spp. were mixed, and amplified products were visualized by hybridizing to dipstick DNA chromatography. The multiplex LAMP with dipstick DNA chromatography distinguished amplified *Rickettsia* and *Plasmodium* targeted genes simultaneously. The determined sensitivity using synthetic nucleotides was 1000 copies per reaction for mixed *Rickettsia* and *Plasmodium* genes. When genomic DNA from in vitro cultured organisms was used, the sensitivity was 100 and 10 genome equivalents per reaction for *Rickettsia monacensis* and *Plasmodium falciparum*, respectively. Although further improvement will be required for more sensitive detection, our developed simultaneous diagnosis technique will contribute to the differential diagnosis of undifferentiated febrile illness caused by either SFG *Rickettsia* spp. or *Plasmodium* spp. in resource-limited endemic areas. Importantly, this scheme is potentially versatile for the simultaneous detection of diverse infectious diseases.

## 1. Introduction

Undifferentiated febrile illnesses (UFIs) are the most common clinical presentation at healthcare centers in low- and middle-income countries [1,2]. With the decline in global malaria incidence, other infectious causes of febrile illness presumptively misdiagnosed as malaria have been identified [3,4,5]. Rickettsioses and malaria are among the common cause of acute UFI, with the disease distribution partly overlapping [6,7]. The acute undifferentiated febrile manifestation leads to challenges in the differential diagnosis of the definitive cause of acute UFI, mostly in incapacitated diagnostic settings. Most clinical diagnostic approaches tend to specifically target more common diseases, such as malaria, hence missing out on other pathogens, including rickettsiae [4,6].

Malaria is still a common cause of febrile illness and death worldwide. The five *Plasmodium* spp. that infect humans and cause malaria include *Plasmodium falciparum*, *P. vivax*, *P. ovale*, *P. malaraie*, and *P. knowlesi.* Sub-Saharan Africa carries almost 80% of the global malaria burden, with *P. falciparum* being the predominant causative agent. Effective management of malaria depends on accurate diagnosis as misdiagnosis can result in significant morbidity and mortality [8]. The current gold standard for malaria diagnosis is microscopy, with more feasible rapid diagnostic tests (RDTs) becoming commonly used. Spotted fever group (SFG) rickettsioses are neglected diseases and represent a large proportion of emerging and re-emerging infectious diseases with a worldwide distribution. Rickettsiosis caused by *Rickettsia africae* causes a significant proportion of acute febrile illnesses among returning travelers from sub-Saharan Africa [9]. *Rickettsia felis* is also a common cause of febrile illness in sub-Saharan Africa, with reported co-infection with *Plasmodium* spp [7]. Other SFG rickettsiae associated with fever in sub-Saharan Africa include *Rickettsia conorii*, *R. sibrica*, *R. massiliae,* and *R. aeschlimannii* [10]. Diagnosis of SFG rickettsiae is based on serological and nucleic acid detection methods, which have limitations depending on the time since infection and sample collection [11]. Nucleic acid detection methods are mostly utilized at the onset of illness, while serological methods are more appropriate after antibody response, which may be late for disease management [12]. The co-infection of rickettsiosis and malaria in humans has been reported in sub-Saharan Africa [7]. Additionally, many cases of non-malarial febrile illnesses have been reported to be attributed to SFG rickettsiae infection [13,14]. As such, differential molecular tests of SFG *Rickettsia* spp. and *Plasmodium* spp. are warranted to correctly diagnose infectious causes of febrile illness in low-resource settings. Therefore, the utilization of molecular methods for multiplex detection of malaria and SFG rickettsia is inevitable.

Nucleic acid amplification tests (NAATs) have been widely used to detect pathogens, including malaria parasites [15,16] and SFG rickettsiae [17,18], of which PCR-based methods have been the mainstay method. However, PCR-based methods are not readily available in resource-limited endemic areas for routine diagnostic procedures because they require expensive and specialized instruments, such as thermocyclers, for accurate temperature fluctuation during amplification. Loop-mediated isothermal amplification (LAMP) is one of the promising alternatives, which is a rapid, simple, and highly specific DNA amplification technique at a constant temperature using DNA polymerases with strand displacement activity [19]. LAMP methods for the detection of *Plasmodium* spp. [20,21,22] and SFG rickettsiae [23,24] have been developed. Despite the high sensitivity of the developed LAMP systems, they can only detect an individual target due to the use of turbidity and/or fluorescence for visualization [25]. Since the amplified LAMP product shows a laddering pattern, the size differentiation by the gel electrophoresis is impossible. For the multiplex LAMP assay, which is aimed at the simultaneous detection of multiple DNA targets in a single reaction, the amplified products can be distinguished by melting curve analysis [26] or DNA lateral flow dipstick techniques [27]. Many other multiplexing LAMP techniques have also been developed using oligo fluorophores and quencher-based probes to differentiate the multiple simultaneous detected amplicons [28,29,30]. However, all these fluorescence-based methods require special equipment, which is often expensive, preventing its use in resource-limited laboratories.

DNA lateral flow dipstick is a DNA detection technique by using DNA-DNA hybridization of a free single strand of amplicons to their complementary probes in the dipstick without amplicon denaturation, and the reaction can be visualized by colorant accumulation [31,32]. Single-stranded tag hybridization chromatographic printed-array strip (C-PAS) is one of the DNA lateral flow dipstick technology designed by Tohoku Bio-Array Company Limited (Miyagi, Japan) [33]. The technique was initially optimized for PCR products and allows discrimination of multiple amplified products simultaneously. The multiple targets amplified with biotin-labeled primer and unique single-stranded sequence tagged primer are developed on C-PAS with streptavidin-coated blue latex beads. The amplified DNA targets with a single-stranded unique tag sequence hybridize to the complementary tag sequence printed on the C-PAS and will show a blue line due to the accumulation of blue latex beads indicating a positive result. Multiple single-stranded unique tag sequences can be used in the multiplex reaction. The specific complementary tag sequences on the chromatographic strip will discriminate the amplified products by hybridizing to the unique tag sequences attached to amplification primers [32,34]. The C-PAS method enables easy differentiation and visualization of multiplex DNA signals in a single tube within a short time (15 min). The simplicity of the C-PAS visualization method makes it suitable for easy visualization of multiple amplified LAMP products. Multiplex LAMP amplification and visualization with C-PAS has been applied in the food industry [27,35] but has not been utilized for differential and simultaneous detection of infectious diseases.

In this study, we aimed at establishing a simple and rapid diagnostic method that could simultaneously detect SFG rickettsiae and *Plasmodium* spp., using optimized multiplex LAMP and C-PAS. The established system can be applied in point-of-care diagnostics and will improve the identification of UFI, especially in resource-poor settings. The concept can also be utilized in other clinically or epidemiologically related infectious diseases.

## 2. Materials and Methods

### 2.1. Multiplex LAMP

The LAMP primers were synthesized based on the previously reported LAMP assays targeting the 17kDA protein-encoding gene of SFG rickettsiae [23] and mitochondrial genomic DNA of human infective *Plasmodium* spp. [22]. The multiplex LAMP reaction consisted of two sets of six LAMP primers (total of 12 primers). The LAMP primer sets consisted of a 5′-terminal modified forward loop primer (LF) for each target template, which was tagged with a carbon spacer and a unique tag sequence complementary to the specific oligos on the C-PAS. The backward loop primer (LB) and backward inner primer (BIP) targeting SFG rickettsiae and *Plasmodium* species, respectively, were labeled with biotin at the 5′-terminal (Tohoku Bio-Array, Miyagi, Japan). All the other primers were not modified (Table 1).

The LAMP mixture was basically the same as the previously reported condition [36,37], except the primer concentration was a quarter for each primer set to avoid primer dimer formation. We optimized the concentration of primers experimentally so that the assay showed the same sensitivity with original primer sets without primer dimer formations. Each 25-µL LAMP reaction mix consisted of 0.25 µL of each primer, resulting in a final concentration of 0.4 µM FIP, 0.4 µM BIP, 0.2 µM LB, 0.2 µM LF, 0.05 µM F3, and 0.05 µM B3 of each LAMP system primer set. Other reagents in the reaction mix were 2.25 µL LAMP reaction buffer (consisting of 1 mM Tris-HCl (pH 10), 1 mM KCl and 1 mM (NH_4_)_2_SO_4_ in 0.1% TritonX-100), 1 µL of Colori-Fluorometric Indicator (CFI; consisting of 3 mM hydroxylnaphthol blue [MP Biomedicals, Aurora, OH, USA]) and 0.35% v/v GelGreen (10,000X solution in DMSO, Biotium, Hayward, CA, USA) in distilled water) [36], 2 µL of 2 M Trehalose solution, 1.4 µL of 25 mM each deoxyribonucleotide triphosphates (dNTPs) (Nippon Gene, Tokyo, Japan), 1 µL of 8 U/μL *Bst* 2.0 WarmStart DNA polymerase (New England Bio Labs Inc.,Ipswich, MA, USA), 1.5 µL of 100 mM MgSO_4_, and 10.85 µL of distilled water with 0.1% Triton X-100. The reaction mix was prepared in the cleanroom and then transferred to the amplification room, where 2 μL of template were added.

The LAMP reactions were performed at 62 °C for 60 min and monitored using the Rotor-Gene 3000 thermocycler FAM channel, which detected the fluorescence of the GelGreen in the LAMP reagents (Corbett Research, Sydney, Australia), and then melting curve analysis was performed.

### 2.2. Visualization of Multiplex Amplified Products by Dipstick DNA Chromatography C-PAS

The C-PAS F4-V2 membrane strip (Tohoku Bio-Array) was inserted into a 21-μL reaction mix containing 10 μL of developing solution (300 mM NaCl) (Tohoku Bio-Array), 9 μL distilled water, 1 μL of LAMP product, and 1 μL of streptavidin-coated blue latex suspension (Tohoku Bio-Array). The band was observed after 15 min at ambient condition (20–25 °C and 30–40% humidity). The blue line on the C-PAS strip test position indicated a positive test result for the presence of the amplified target DNA sequence tagged with the complementary oligonucleotide and biotin. LAMP amplification and the visualization with dipstick chromatography was performed in the post-amplification room, which is independent from the pre-amplification clean room.

### 2.3. The Determination of the Sensitivity of Multiplex LAMP Assay Using Synthetic Genes

The pEX-A2J2 plasmid carrying the synthetic 361 bp of 17kDa protein-encoding gene of *R. felis* (accession number CP000053), which is conserved among SFG rickettsiae, was obtained from the company (Eurofins Genomics, Tokyo, Japan). This template was referred to as *Rickettsia* gene in this manuscript. DNA fragment (361 base pair) of the pan-*Plasmodium* genus was also inserted into the plasmid based on the *P. falciparum* mitochondrial sequence (accession number AJ276844), thereby referred to as the *Plasmodium* gene. The Qubit 4.0 fluorometer dsDNA HS assay kit (Thermo Fischer Scientific, Waltham, MA, USA) was used to quantify the concentration of the plasmids. The templates were normalized to equal copy numbers and mixed in equal proportions. The mixed template was then serially diluted by 10-fold every time before use. The serially diluted copy number ranged from 10 to 1x10^6^ gene copies per µL. The assays were performed in five replicates for each template concentration, with five non-template controls.

### 2.4. The Determination of the Sensitivity of Multiplex LAMP Assay Using Genomic DNA from In Vitro Cultured R. monacensis or P. falciparum

*Rickettsia monacensis*, one of the SFG rickettsiae, was maintained in the C6/36 cell line described in a previous study [38]. Genomic DNA was extracted from the cultured *R. monacensis* by Takara Simpleprep kit (Takara, Shiga, Japan) and purified by ethanol precipitation. The *R. monacensis* genomic copy number was then quantified by real-time qPCR using a single copy SFG-specific *OmpA* gene using previously described primers [39]. DNA quantification was determined using the Bio-Rad CFX Manager software based on the standard curve. The determined DNA copy number was assumed to be equivalent to the genomic copy number (genome equivalents). *P. falciparum* HB3 strain was cultured in vitro according to Trager and Jensen [40] at 0.1–1.0% parasitemia. The genomic DNA was extracted using a Nucleospin blood kit (Takara). The obtained DNA concentration was then measured by the dsDNA HS assay kit with a Qubit 4.0 fluorometer. The DNA quantities were converted to the genome equivalents by taking the *Plasmodium* genome size to be 22.9 Mb. The genomic DNA of in vitro cultured *R. monacensis* and *P. falciparum* were 10-fold serially diluted. The multiplex LAMP was tested using the templates as above. The same templates were also used for comparative sensitivity analysis of multiplex LAMP and real-time qPCR. The previously reported *OmpA* [39] and 18S rRNA [41] gene-based qPCR was tested on the *R. monacensis* and *P. falciparum* genomic DNA, respectively. In brief, the SYBR green-based qPCR was performed in the CFX96 Touch Real-Time PCR Detection System (Bio-Rad, Hercules, CA, USA). All reaction mixtures had a final volume of 20 μL with 12.5 μL of TB Green Premix Ex Taq II (Takara, Japan), 1 μL (0.4 μM final concentration) of each primer, and 2 μL of quantified genomic DNA template. After the initial denaturation cycle at 95 °C for 30 s, reactions were cycled 40 times as follows: 95 °C for 5 s and 50 °C for 30 s with a final melting curve step.

### 2.5. Detection of R. monacensis and P. falciparum Spiked with Human Blood from DNA Card Using Heating Block and Dipstick Chromatography

*Rickettsia monacensis* from in vitro culture was spiked with human blood at 5000 bacteria copies per µL of blood. The *Rickettsia* spiked with blood was then spotted on the classic Whatman FTA Classic card (GE Healthcare, Buckinghamshire, UK) and dried. Similarly, red blood cells infected with *P. falciparum* at 1% parasitemia were dried on the Whatman FTA Classic card (GE Healthcare, UK). A negative template control was prepared by applying uninfected blood onto the Whatman FTA Classic card (GE Healthcare, UK) and dried it. The DNA from dried blood spots was extracted by the modified boiling method [42]. In brief, two dried blood spots of 3 mm were punched out and soaked in 40 µL of nuclease-free water in the PCR tube. The mixed template was prepared by mixing two dried blood spot punches from *R. monacensis* and *P. falciparum* in a single tube. The samples were then incubated at 60 °C for 30 min and boiled at 99 °C for 10 min. The multiplex LAMP was performed on a heating block, powered by a portable rechargeable battery, at 62 °C for 60 min. The amplicon was then visualized using a portable fluorometer [36] and dipstick chromatography.

## 3. Results

### 3.1. Multiplex LAMP Using Individual Synthetic Templates of Rickettsia or Plasmodium Genes in Plasmids

**Figure 1:**
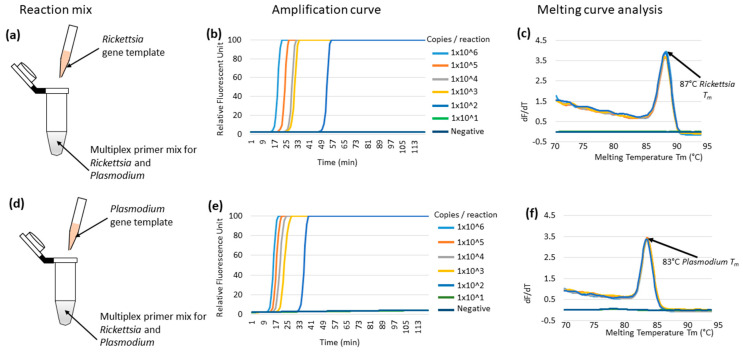
Multiplex LAMP amplification using a synthetic single target gene template. (**a**) shows the multiplex LAMP primer mix and *Rickettsia* gene template. The amplification curve in (**b**) shows the detection of up to 100 *Rickettsia* gene copies per reaction. The melting temperature for *Rickettsia* gene is shown to be 87 °C in (**c**). (**d**) shows the multiplex primer mix and the *Plasmodium* gene template for the reaction mix. The multiplex LAMP sensitivity shows the detection of up to 100 *Plasmodium* gene copies per reaction (**e**). The *Plasmodium* gene melting temperature shows 83 °C (**f**).

The reported two LAMP primer sets targeting *Rickettsia* 17kDa protein-encoding gene [23] and *Plasmodium* mitochondrial DNA [22] were utilized for multiplex LAMP reaction. We first tested the multiplex LAMP reaction by mixing two primer sets at a 25%, 50%, and 100% quantity from the original condition. Since the 50% and 100% primer quantity produced nonspecific amplification, most possibly by primer dimerization, we decided to use the 25% each primer concentration for multiplexing. The selected 25% primer concentration did not affect the detection sensitivity, although a delay of the amplification time was observed when compared with the 100% primer concentration (Supplementary Materials Figure S1). The target loci for the two LAMP reactions were synthesized and inserted in the plasmids. Using this condition, the multiplex LAMP successfully detected up to 100 template copies per reaction using either the *Rickettsia* or *Plasmodium* gene in individual reaction tubes (Figure 1b,e). The amplification was monitored by a real-time PCR machine, and its specificity was confirmed by melting curve analysis [43]. The melting temperature (Tm) for the *Rickettsia* and *Plasmodium* gene was 87 and 83 °C, respectively (Figure 1c,d, respectively). Based on this result, we concluded that the SFG rickettsiae and *Plasmodium* spp. LAMP systems were compatible for multiplexing.

### 3.2. Optimization of Multiplex LAMP Using Mixed Synthetic Templates of Rickettsia and Plasmodium Gene, and Discrimination by C-PAS

**Figure 2:**
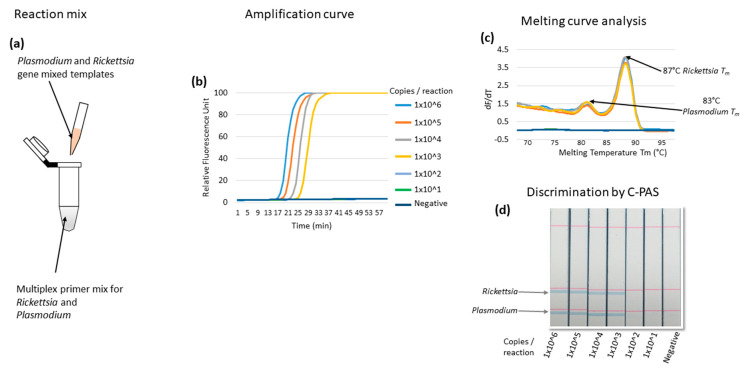
Multiplex LAMP amplification and discrimination by dipstick DNA chromatography (C-PAS) using mixed target gene templates. The reaction mix contained multiplex LAMP primer sets and the two-template mix for *Rickettsia* and *Plasmodium* gene target templates (**a**). The amplification curve detected up to 1000 indistinguishable gene copies per reaction (**b**). The melting curve analysis panel shows two melting temperatures for *Rickettsia* and *Plasmodium* genes at 87 and 83 °C, respectively (**c**). (**d**) shows the dipstick DNA chromatography panel, indicating *Rickettsia* and *Plasmodium* genes, amplified products distinguished as two bands. The C-PAS result is consistent with the melting curve analysis.

To test if the multiplex LAMP could amplify multiple targets simultaneously in a single reaction, equally mixed templates of *Rickettsia* and *Plasmodium* genes were amplified, and discrimination by C-PAS was tested. The amplification efficiency was observed by fluorescence by real-time PCR machine monitoring, but multiple target amplified products cannot be distinguished based on amplification fluorescence (Figure 2b). The simultaneously amplified products of *Rickettsia* and *Plasmodium* target gene were first discriminated by melting curve analysis. Two melting temperatures were observed at 87 and 83 °C, indicating successful simultaneous amplification of *Rickettsia* and *Plasmodium* target genes (Figure 2c). The simultaneously amplified products were further discriminated by C-PAS, which showed a consistent result as the melting temperature analysis. According to the results, the multiplex LAMP amplified both targets, and the simultaneously amplified products were distinguishable by C-PAS (Figure 2d). The multiplex LAMP for the simultaneous detection of both *Rickettsia* and *Plasmodium* genes detected up to 1000 copies of each template per reaction. The discrimination of mixed *Rickettsia* and *Plasmodium* genes amplified by multiplex LAMP was consistently comparable for C-PAS and melting curve analysis in five independent experiments. The non-template controls were consistently negative in all assays (Table 2). This result showed the potential of C-PAS to be applied for distinguishing multiplex amplified products without using a thermocycler.

### 3.3. Validation of Multiplex LAMP Using Genomic DNA from In Vitro Cultured R. monacensis or P. falciparum Organisms

**Figure 3:**
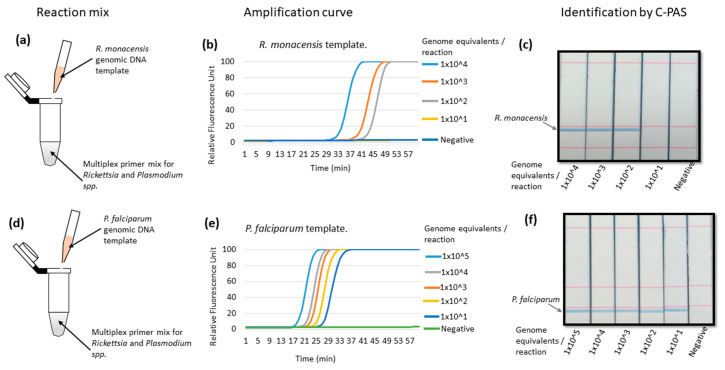
Multiplex LAMP amplification using genomic DNA from in vitro cultured organisms. (**a**) shows the multiplex LAMP primer sets with *R. monacensis* genomic DNA in the reaction mix. The amplification curve shows detection up to 100 genome equivalents per reaction of *R. monacensis* (**b**) and identification of amplicons as *Rickettsia* by C-PAS (**c**). The reaction mix contained multiplex LAMP primer sets with *P. falciparum* genomic DNA (**d**). (**e**) shows the amplification curve of *P. falciparum* detecting up to 10 genome equivalents per reaction. C-PAS identified the amplified product as *Plasmodium* (**f**).

The multiplex LAMP amplification was further tested with genomic DNA from the individual target of in vitro cultured *R. monacensis* or *P. falciparum* organisms as reaction templates (Figure 3a,d). Multiplex LAMP detected up to 100 genome equivalents of *R. monacensis* per reaction and was identifiable on C-PAS (Figure 3b,c). When genomic DNA from *P. falciparum* was used, multiplex LAMP detected up to 0.26 pg, which was equivalent to 10 genome per reaction, and was also identifiable on C-PAS (Figure 3e,f). The observed 10 genome equivalents detection limit was greater than the result from the synthetic gene (Figure 2b–e) because *Plasmodium* LAMP targets mitochondrial cytochrome *b* gene, which has 20–120 copies in one parasite depending on its lifecycles [44].

### 3.4. Comparative Sensitivity of the Multiplex LAMP System against the Real-Time qPCR System

**Figure 4:**
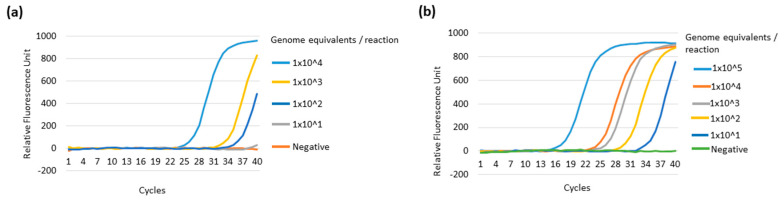
The sensitivity of real-time qPCR using genomic DNA from in vitro cultured organisms. (**a**) shows the amplification curve of *R. monacensis* by qPCR based on the *Rickettsia OmpA* gene. (**b**) shows the *Plasmodium* 18S rRNA gene amplification curve using the *P. falciparum*. The qPCR showed detection up to 100 (**a**) and 10 (**b**) genome equivalents for in vitro cultured *R. monacensis* and *P. falciparum,* respectively.

The multiplex LAMP performance was compared to the reported real-time qPCR system using a single target genomic DNA of either in vitro cultured *R. monacensis* or *P. falciparum*. The real-time qPCR targeting the SFG *Rickettsia* outer membrane protein A (*OmpA*) gene detected up to 100 genomic DNA copies per reaction, indicating that our multiplex LAMP was comparable to the real-time qPCR method (Figure 3b and Figure 4a). Similarly, real-time qPCR targeting pan-*Plasmodium* 18S rRNA detected up to 10 genome equivalents (0.26 pg), showing the same sensitivity with our multiplex PCR system (Figure 3e and Figure 4b).

### 3.5. Simplified LAMP Reaction Using Human Blood Spiked Samples and Field-Deployable Amplification Methods

**Figure 5:**
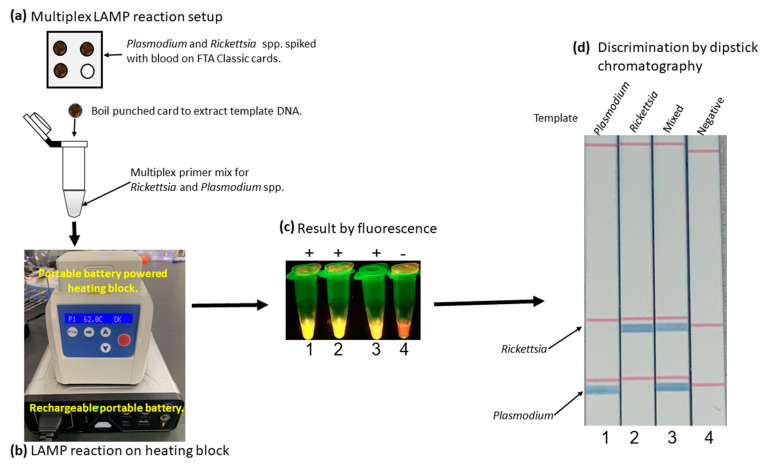
Simplified multiplex LAMP detection of *R. monacensis* and *P. falciparum* spiked with human blood. (**a**) shows the multiplex LAMP reaction setup using *R. monacensis* and *P. falciparum* spiked with blood, dried on Whatman FTA Classic card, and genomic DNA extracted by boiling. (**b**) shows the performance of LAMP using a rechargeable battery-powered heating block. (**c**) shows the detection result by fluorescence, while (**d**) shows multiple amplicon discrimination by dipstick chromatography.

To test the developed method’s applicability in a simple field condition, the multiplex LAMP was performed on the heating block powered by a portable battery using cultured parasites spiked with normal human blood. The multiplex LAMP successfully amplified *R. monacensis* and *P. falciparum* (Figure 5a,b). The amplified product was visualized by the fluorescent detection method using a portable fluorometer (Figure 5c). The amplified products were further confirmed to the detected species by dipstick chromatography. The test clearly distinguished the amplified *Rickettsia* spp. and *Plasmodium* spp. as well as simultaneous amplification. The negative control using genomic DNA extracted from known negative blood was negative, indicating no nonspecific amplification (Figure 5d).

## 4. Discussion

Differential diagnosis of the diseases with similar clinical manifestation is critical for successful case management outcomes. Spotted fever group rickettsia, such as *R. felis,* has been reported to co-infect febrile patients with *Plasmodium* spp. in endemic areas [7]. Co-infection of *Plasmodium* spp. with other pathogens, such as dengue virus and *Leptospira* spp., is associated with severe malaria [45]. Other rickettsial infections have been reported to be mixed with either *Plasmodium*, *Leptospira,* or *Coxiella burnetii*, resulting in complicated clinical management and poor prognosis [46,47,48,49]. Therefore, the development of multiplex differential diagnostic tools is essential for improved diagnosis and case management of undifferentiated fevers.

Our established multiplex LAMP system can be applied in both differential and simultaneous detection of SFG rickettsiae and *Plasmodium* spp. The multiplex LAMP primer design was adopted from the previously described LAMP systems [23,50], suggesting that already developed LAMP systems could be improved by multiplexing. This proof of concept shows that LAMP methods can be multiplexed to detect infectious diseases and discriminate by dipstick DNA chromatography (C-PAS) visualization, hence easing their application as a point-of-care differential diagnostic tool. The use of C-PAS makes it possible to differentiate multiple amplified targets and visualize without any sophisticated equipment, such as a thermocycler, for melting curve analysis. Since LAMP can also be performed on a heating block or water bath for amplification without a thermocycler [51,52], the whole procedure of our established multiplex LAMP and C-PAS requires minimal resources. The differentiation and visualization of multiple amplified products by C-PAS, which shows multiple blue lines for multiple amplified products, allows easy differential diagnosis in clinical settings. However, it is recommended to use different rooms to prepare the multiplex LAMP master mix and perform dipstick chromatography where the LAMP reaction tube is opened to avoid cross-contamination. Herein, we demonstrated the multiplexing of SFG rickettsia and pan-*Plasmodium* LAMP and distinguished the specific amplicons by C-PAS. This technique can also be applied in various differential diagnoses. For example, multiplexing of *P. falciparum* and non-*P. falciparum* LAMP could be beneficial for their differentiation as artemisinin tends to fail to clear non-*P. falciparum* malaria [53].

Multiplex LAMP was a combination of two LAMP primer sets in the same reaction. The target template was either mixed (two) or single (one) target templates. The multiplex LAMP’s sensitivity on the mixed targets was 10 times (1000 copies/reaction) lower than the single target template (100 copies/reaction). The discrepancy could be attributed to the competitiveness of multiplex reactions for reagents in mixed target template reactions. However, the multiplex LAMP is potentially applicable in clinical diagnosis as the malaria parasitemia was reported to be approximately 1000 parasites per µL [44,54], translating into detectable quantities by the multiplex LAMP system with dipstick DNA chromatography. SFG rickettsia pathogens, such as *R. rickettsii*, have shown bacteremia of more than 100 copies per µL in fatal cases [55], which can be detected by our multiplex LAMP.

## 5. Conclusions

In summary, a multiplex LAMP system to simultaneously detect SFG rickettsiae and pan-*Plasmodium* spp was developed. The differentiation of the multiple amplified targets was accomplished by dipstick DNA chromatography (C-PAS). Our results show that LAMP can be multiplexed for the simultaneous detection of infectious diseases and easily visualized by dipstick DNA chromatography. Multiplex LAMP is simple and can be applied at a resource-limited point of care as it does not require sophisticated equipment and simple analysis for multiple pathogen diagnosis. Nevertheless, the multiplex LAMP method still needs further improvements on its sensitivity for its application in non-fatal clinical cases with low parasitemia.

## Figures and Tables

**Table 1:** Loop-mediated isothermal amplification primer sequences that were used for multiplex LAMP. Tagged and biotin-labeled primers are shown with F1 and F4, representing a unique tag sequence. “X” indicates the position of spacer C3 between the unique tag sequence and the LAMP primer sequence.

Target Pathogen	Target Gene Name	Primer Name [Reference]	Primer Sequence Name	Primer Sequence
Spotted fever and Transitional group rickettsiae	17 kDa protein-encoding (*htrA*) gene	17 kDA [23]	Rr17F3	5′-TGTTACAAGCCTGTAACGG-3′
			Rr17B3	5′-TCCTGTTCATCCATACCTG-3′
			Rr17FIP	5′-GAGAACCAAGTAATGCGCCGGGCGGTATGAATAAACAAGG-3′
			Rr17BIP	5′-AATTCGGTAAGGGCAAAGGACCACCGATTTGTCCACCAA-3′
			Rr17LoopF	5′-F1-X-CCGCCAAGAAGTGTTCCTGTA-3′
			Rr17LoopB	5′-Biotin-AGCTTGTTGGAGTAGGTGTAGGTG-3′
*Plasmodium* spp.	Mitochondrial *Cytochrome b* (*cytb*) gene	PgMt19 [22]	PgMt19-F3	5′-TCGCTTCTAACGGTGAAC-3′
			PgMt19-B3	5′-AATTGATAGTATCAGCTATCCATAG-3′
			PgMt19-FIP	5′-GGTGGAACACATTGTTTCATTTGATCTCATTCCAATGGAACCTTG-3′
			PgMt19-BIP	5′-Biotin-GTTTGCTTCTAACATTCCACTTGCCCGTTTTGACCGGTCATT-3′
			PgMt19-LF	5′-F4-X-CACTATACCTTACCAATCTATTTGAACTTG-3′
			PgMt19-LB	5′-TGGACGTAACCTCCAGGC-3′

**Table 2:** The sensitivity of the multiplex LAMP system based on the melting curve analysis and C-PAS detection technique. Melting curve analysis and C-PAS showed the same sensitivities for detecting synthetic genes of *Plasmodium* and *Rickettsia* in five independent experiments. All experiments were performed in five replicates. NTC: no template control.

Copy Number Per Reaction		1,000,000	100,000	10,000	1000	100	10	NTC
Melting peak analysis result	*Plasmodium* gene	5/5 (100%)	5/5 (100%)	5/5 (100%)	5/5 (100%)	3/5 (60%)	1/5 (20%)	0/5 (0%)
	*Rickettsia* gene	5/5 (100%)	5/5 (100%)	5/5 (100%)	5/5 (100%)	0/5 (0%)	0/5 (0%)	0/5 (0%)
C-PAS result	*Plasmodium* gene	5/5 (100%)	5/5 (100%)	5/5 (100%)	5/5 (100%)	3/5 (60%)	1/5 (20%)	0/5 (0%)
	*Rickettsia* gene	5/5 (100%)	5/5 (100%)	5/5 (100%)	5/5 (100%)	0/5 (0%)	0/5 (0%)	0/5 (0%)

## Supplementary Materials

The following are available online at https://www.mdpi.com/2075-4418/10/11/897/s1, Figure S1. Optimization of the primer concentration for multiplexing.
