# Supplementary material for: Development of a Multiplex Loop-Mediated Isothermal Amplification (LAMP) Method for Simultaneous Detection of Spotted Fever Group Rickettsiae and Malaria Parasites by Dipstick DNA Chromatography
Source: Diagnostics (Basel). 2020 Nov 2;10(11):897. doi: 10.3390/diagnostics10110897 (PMC7694008; doi:10.3390/diagnostics10110897)
Supplement: Supplementary file 1 [file diagnostics-10-00897-s001.pdf]

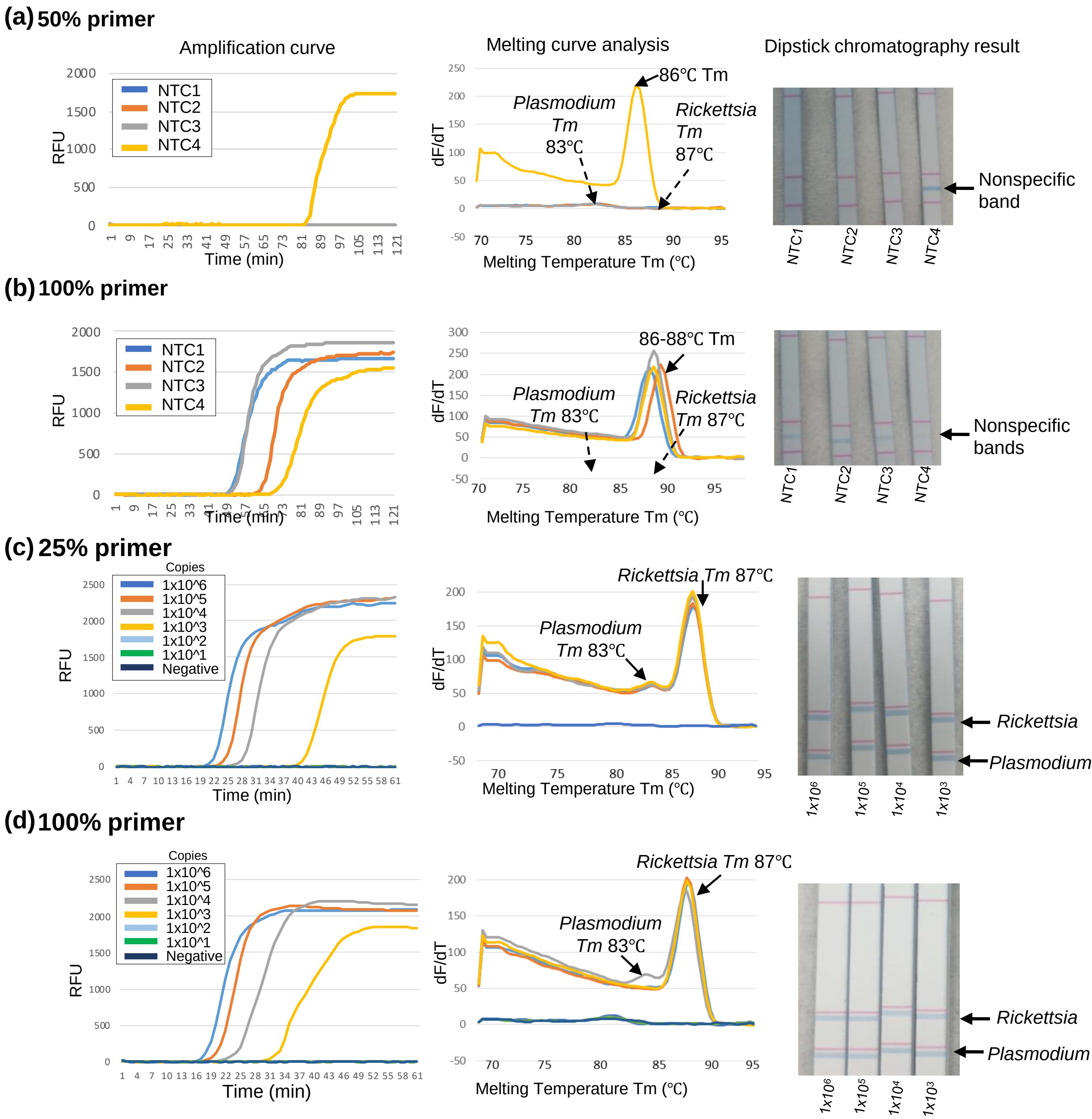

Figure S1. Optimization of the primer concentration for multiplexing.
False positive amplifications were observed in 50% primer concentration (a) and 100% primer concentration (b): At higher primer concentration conditions, 1 of 4 (50% condition) and 4 of 4 (100% condition) resulted in non-specific amplification in no template control (NTC)**.** About 86-88 ℃ Tm value were obtained from these amplification products, suggesting non-specific amplification. Nonspecific band is also observed in the dipstick chromatography at the *Plasmodium* position. Note that this experiment uses different tag F3 for *Plasmodium*, so the location of the band is different from sensitivity assay below.
Comparative sensitivity of 25% (c) and 100% (d) primer concentration: In the optimized primer concentration at 25%, up to 1000 copies were detected both in *Plasmodium* and *Rickettsia*, as also shown in main Figure 2. This detection limit is comparable to the result obtained in 100% primer condition, although delay of the amplification was observed. No false positive amplification was observed in this experiment.
